# Femoral head infarction: a case-control study of a rare complication

**DOI:** 10.1186/s13018-025-06243-8

**Published:** 2025-09-24

**Authors:** Guanying Gao, Rongge Liu, Yichuan Zhu, Jiayang Liu, Jianquan Wang, Yan Xu

**Affiliations:** Department of Sports Medicine, Institute of Sports Medicine, Beijing Key Laboratory of Sports Injuries, Peking University Third Hospital, 49 North Garden Road, Haidian District, Beijing, 100191 China

**Keywords:** Hip arthroscopy, Cam deformity, Femoroacetabular impingement, Femoral head infarction, Postoperative complication

## Abstract

**Background:**

To determine the prevalence of the femoral head infarction (FHI) following hip arthroscopy, explore potential contributing factors, and assess its influence on clinical outcomes.

**Methods:**

We evaluated consecutive patients who underwent hip arthroscopy in our hospital between May 2014 and May 2023 retrospectively. Patients underwent MRI at least one year after surgery. FHI was identified as large scale edema signal at the junction of the femoral head and neck in postoperative MRI. FHI were matched in a 1:3 cohort to the normal group based on sex, age, and body mass index (BMI). Preoperative patient-reported outcomes (PROs) and PROs at least one year after surgery were obtained.

**Results:**

A total of 372 patients were finally included in this study. In the MRI follow-up, it was discovered that ten patients (2.7%) had FHI. Patients in FHI group had significantly lower BMI than patients without FHI (*P* = 0.008). Patients in the FHI group did not show significant improvements in postoperative mHHS, iHOT-12, or VAS scores (*P* > 0.05). Patients in the FHI group demonstrated significantly lower postoperative mHHS and iHOT-12 scores, along with higher VAS scores (*P* < 0.05). In the FHI group, only 3 patients (30%) surpassed the MCID and achieved the PASS for mHHS, while no patients surpassed the MCID or achieved the PASS for iHOT-12. The proportion of patients who achieved the MCID or the PASS in the normal group was significantly higher than that in the FHI group (*P* < 0.05).

**Conclusion:**

Our study demonstrated that FHI could be a rare complication following hip arthroscopy. Patients with lower BMI are at a higher risk of developing postoperative FHI. The clinical outcomes for patients with FHI are poor, with no significant improvement in patient-reported outcomes observed.

## Introduction

Hip arthroscopy has indeed seen significant advancements and a notable rise in utilization [1–8]. However, like any medical procedure, it is not without its risks. With the growing frequency of hip arthroscopy procedures, complications are being observed and reported more frequently [5, 9]. The overall incidence of complications is relatively low, ranging from 1.3 to 4.2% [10–13]. The majority of complications associated with hip arthroscopy are considered minor. These complications may include septic arthritis, neuropraxia, hemorrhage, bursitis, instrument breakage, chondral and labral damage, as well as fluid extravasations [9, 14–16]. Avascular necrosis and femoral neck fracture are typically considered theoretical complications in the excision of an impingement lesion during hip arthroscopy [14, 17]. However, there have been documented cases of these complications reported in the medical literature [18]. In our routine clinical practice, we have identified a previously unreported complication after hip arthroscopy. We have observed that certain patients may exhibit edema signals in magnetic resonance imaging (MRI) at the femoral head-neck junction on postoperative MRI scans, initially suspected to be femoral head infarction (FHI). Given the absence of prior research documenting this complication, further investigation is warranted to elucidate and understand this phenomenon.

The purpose of this study is to determine the prevalence of the identified complication, explore potential contributing factors, and assess its influence on clinical outcomes. It is hypothesized that FHI occurs at a specific rate after hip arthroscopy and may have negative influence on the overall clinical outcomes of patients who underwent hip arthroscopy.

## Methods

### Patients

We evaluated consecutive patients who underwent hip arthroscopy in our hospital between May 2014 and May 2023 retrospectively. The inclusion criteria were as follows: (1) patients who underwent hip arthroscopy for femoroacetabular impingement (FAI) in our hospital; (2) had preoperative X-rays, computer tomography (CT) and MRI; and (3) had postoperative MRI at least one year after surgery. The exclusion criteria were as follows: (1) previous hip surgery, (2) avascular necrosis of the femoral head, (3) Legg-Calve-Perthes disease, (4) Ehlers–Danlos syndrome, (5) pigmented villonodular synovitis, osteoid osteoma, synovial chondromatosis, and rheumatologic disease. Patients with postoperative FHI were matched in a 1:3 cohort to the normal group (patients who underwent hip arthroscopy treating FAI but did not have postoperative FHI) based on sex, age, and body mass index (BMI). The study was approved by the Ethics Committee of our hospital.

### Surgical procedure

All arthroscopic surgeries were performed by a single surgeon with more than 15 years’ experience. All surgeries were performed with the patient under spinal anesthesia in the modified supine position as described in previous study [19]. In brief, the interportal capsulotomy technique was used to access the hip joint after establishing the anterolateral and mid-anterior portals. A detailed inspection of the central compartment was performed to assess the acetabular rim, acetabular labrum, articular cartilage, and ligamentum teres. Labral repair or labral debridement was performed according to the nature of the injury. Femoral osteoplasty or acetabuloplasty was performed according to the intraoperative findings. Capsular closure was routinely done at the end of surgery.

### Radiographic assessment

Anteroposterior hip radiographs, Dunn view radiographs, and computed tomography (CT) scans were conducted for all patients both before and after the surgical procedure. Lateral center-edge angle (LCEA) and alpha angle of the hip joint were measured preoperatively and postoperatively. The alpha angle and LCEA were measured from CT and radiographic images using the method described by Notzli et al. [20] and Ömeroglu et al. [21]. Postoperative MRI was performed for radiological follow-up, especially for those who did not achieve great improvement after hip arthroscopy in our institution. Patients underwent MRI at least one year after surgery. FHI was identified as large-scale T1 hypointense and T2 hyperintense signals at the junction of the femoral head and neck in postoperative MRI. One patient underwent MRI reconstruction to enhance visualization of the FHI location. The MRI three-dimensional (3D) reconstructions were conducted using Mimics software version 21.0, based on postoperative MRI scans. Initially, the mask of the target object was delineated slice by slice. Subsequently, the created masks were refined using the erase and draw tools to differentiate the ischemic region from the normal femoral head region. Finally, high-quality objects were generated and used for measuring the volume, height, and length of the ischemic region.

### Clinical evaluation

Preoperative patient-reported outcomes (PROs) and PROs at least one year after surgery were obtained in FHI group and normal group, including visual analog scale (VAS) for pain, International Hip Outcome Tool (iHOT-12) and modified Harris Hip Score (mHHS). Due to the patient’s personal reasons and limited medical resources, the time for MRI follow-up is not exactly the same as that of PROs. For the mHHS, the minimal clinically important difference (MCID) was defined as 8 by Kemp et al. [22], and the patient acceptable symptom state (PASS) score was defined as 74 by Chahal et al. [23]. For iHOT-12, the MCID and PASS were defined as 13.9 and 72.2 by Benedict et al. [24].

### Statistical analysis

Continuous variables were described using the mean ± standard deviation (SD), and categorical data were expressed as a percentage (%). Discrete variables were shown as medians accompanied by interquartile range and were compared with the Mann-Whitney U test. Continuous variables with a normal distribution in the baseline data between groups were examined using the independent-samples t test. Percentages were compared using the chi-square test. Propensity score matching method was used in the matching of the FHI group and the normal group. *P* value of < 0.05 was considered to indicate statistically significant differences. All statistical analyses were carried out using SPSS v27 (IBM Corporation, Armonk, NY, USA).

## Results

**Fig. 1:**
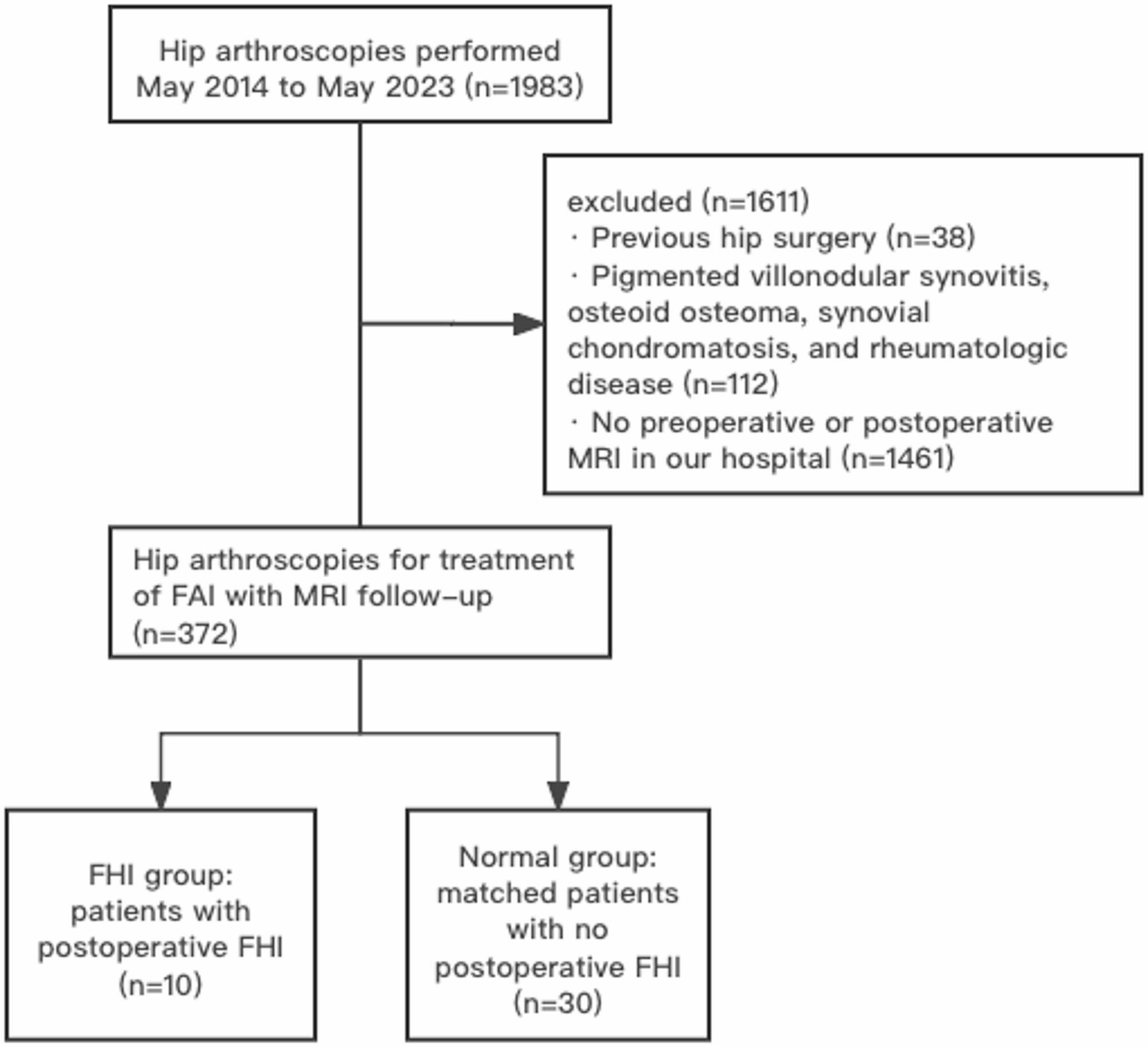
Flowchart illustrating patient selection process. **Note**. This figure showed the selection of participants in this study

A total of 1983 patients who underwent hip arthroscopy in our hospital were evaluated. A total of 372 patients (167 men and 205 women) were finally included in this study. A flowchart illustrating the full patient selection process can be found in Fig. 1. In the MRI follow-up, it was discovered that ten patients (2.7%) had FHI. Patients in FHI group had significantly lower BMI than patients without FHI (*P* = 0.008).

### FHI, femoral head infarction; FAI, femoroacetabular impingement

**Fig. 2:**
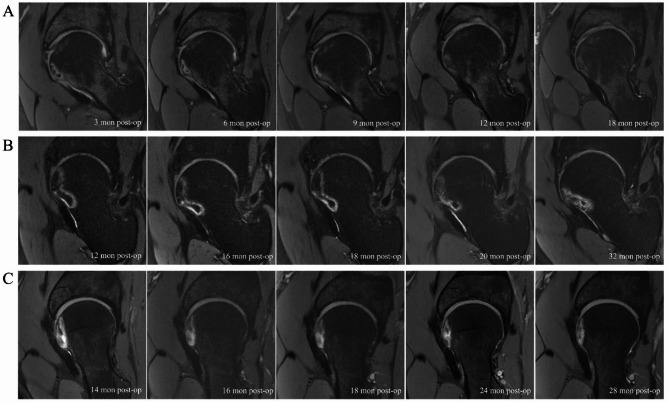
MRI assessment of patients with subsequent follow-ups. **Note**. Dynamic MRI changes in three patients identified with FHI during MRI follow-up. **A-C** represents three different patients. FHI, femoral head infarction; MRI, magnetic resonance imaging

**Fig. 3:**
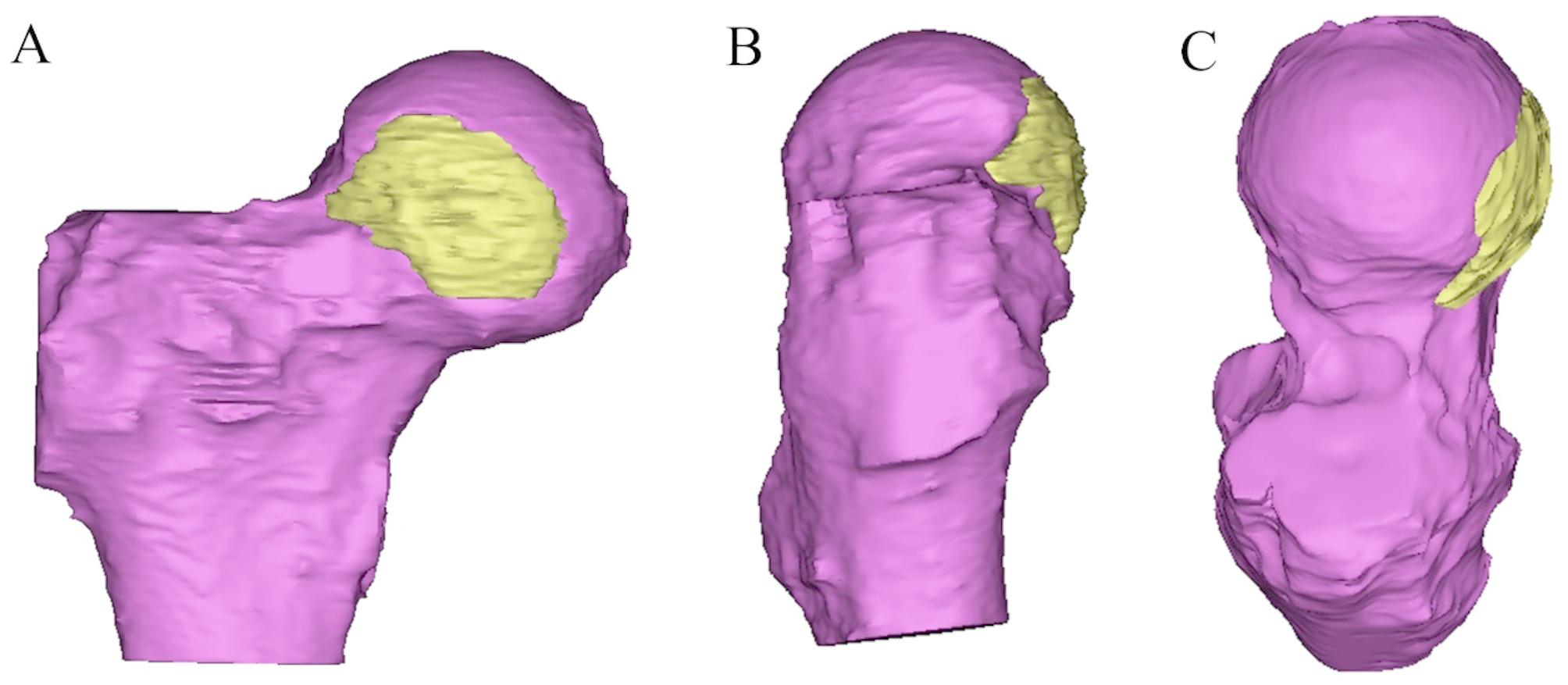
MRI reconstruction of FHI area. **Note**. One patient underwent MRI 3D reconstruction to enhance visualization of the FHI location. All FHIs were located in the anterior region of the femoral head. (**A**) Viewing from the front. (**B**) Viewing from the lateral side. (**C**) Viewing from above. Yellow area showed the range of FHI. Red area showed the normal femoral head. FHI, femoral head infarction

Out of the ten patients identified with FHI during MRI follow-up, three patients underwent subsequent continuous MRI follow-up (Fig. 2). One patient underwent MRI reconstruction to enhance visualization of the FHI location (Fig. 3). We found that all FHIs were located in the anterior region of the femoral head. Thirty patients were matched based on surgery time, follow-up time, sex, age, and body mass index (BMI) as normal group. (Tables 1 and 2) No significant differences were observed in terms of age, sex, BMI, length of follow-up, diagnosis, alpha angle, and LCEA of patients in the FHI group and normal group (*P* > 0.05). Arthroscopic procedures performed in two groups were shown in Table 3.

**Table 1:** Demography of patients in FHI group and patients without FHI (*n* = 372)

	Patients without FHI	FHI group	*P* value
Number	362	10	NA
Age, years, median (range)	36 (18–60)	38 (31–44)	0.56
Sex			
Male	167 (44.9%)	4 (40%)	
Female	205 (55.1%)	6 (60%)	0.75
Side			
Left	182 (48.9%)	4 (40%)	
Right	190 (51.1%)	6 (60%)	0.58
BMI, Kg/m^2^, mean ± SD	23.0 ± 3.2	19.1 ± 1.2	**0.008**
Diagnosis			
Labral tear	372 (100%)	10 (100%)	0
Cam type FAI	350 (94.1%)	10 (100%)	0.42
Pincer type FAI	275 (73.9%)	7 (70%)	0.78
Mixed type FAI	269 (72.3%)	7 (70%)	0.87
SSI	48 (12.9%)	1 (10%)	0.78
BDDH	38 (10.2%)	1 (10%)	1
Alpha angle, mean ± SD	58.2 ± 9.8	58.8 ± 7.2	0.78
LCEA, mean ± SD	34.1 ± 6.5	33.6 ± 4.5	0.71

Unless otherwise specified, data are numbers of patients, with percentages in parentheses. BMI, body mass index; LCEA, lateral center-edge angle; SSI, subspine impingement; FAI, femoroacetabular impingement; BDDH, borderline developmental dysplasia of the hip; FHI, femoral head infarction

**Table 2:** Demography of patients in FHI group and normal group (*n* = 40)

	Normal group	FHI group	*P* value
Number	30	10	NA
Age, years, median (range)	35 (18–50)	38 (31–44)	0.16
Sex			
Male	12 (40%)	4 (40%)	
Female	18 (60%)	6 (60%)	1
Side			
Left	14 (46.7%)	4 (40%)	
Right	16 (53.3%)	6 (60%)	0.71
BMI, Kg/m^2^, mean ± SD	20.0 ± 1.6	19.1 ± 1.2	0.22
Diagnosis			
Labral tear	30 (100%)	10 (100%)	0
Cam type FAI	28 (93.3%)	10 (100%)	0.40
Pincer type FAI	20 (66.7%)	7 (70%)	0.85
Mixed type FAI	20 (66.7%)	7 (70%)	0.85
SSI	3 (10%)	1 (10%)	1
BDDH	3 (10%)	1 (10%)	1
Alpha angle, mean ± SD	59.2 ± 8.8	58.8 ± 7.2	0.82
LCEA, mean ± SD	39.4 ± 7.6	33.6 ± 4.5	0.77
Length of follow-up, mean ± SD, months	25.1 ± 8.4	29.1 ± 12.4	0.47

Unless otherwise specified, data are numbers of patients, with percentages in parentheses. BMI, body mass index; LCEA, lateral center-edge angle; SSI, subspine impingement; FAI, femoroacetabular impingement; BDDH, borderline developmental dysplasia of the hip; FHI, femoral head infarction. NA, not applicable

**Table 3:** Arthroscopic procedures performed in normal group and FHI group

	Normal group (*n* = 30)	FHI group (*n* = 10)	*P* value
Labral repair	30 (100%)	10 (100%)	0
Femoral osteoplasty	28 (93.3%)	10 (100%)	0.40
Acetabuloplasty	20 (66.7%)	7 (70%)	0.85
Chondroplasty	5 (16.7%)	2 (20%)	0.81
Ligamentum teres debridement	2 (6.7%)	1 (10%)	0.73
Focal subspinal decompression	3 (10%)	1 (10%)	1
Capsular plication	3 (10%)	1 (10%)	1

Data are numbers of patients, with percentages in parentheses. FHI, femoral head infarction

The patient-reported outcome scores (VAS, mHHS, iHOT-12) in the preoperative evaluation and follow-up are presented in Table 4. Patients in the normal group exhibited significant improvements in both preoperative and postoperative mHHS, iHOT-12, and VAS scores (*P* < 0.05). Conversely, patients in the FHI group did not show significant improvements in postoperative mHHS, iHOT-12, or VAS scores (*P* > 0.05). There was no significant difference in the preoperative patient-reported outcome scores between the normal group and the FHI group (*P* > 0.05). However, patients in the FHI group demonstrated significantly lower postoperative mHHS and iHOT-12 scores, along with higher VAS scores (*P* < 0.05).

**Table 4:** Preoperative and postoperative Patient-Reported outcome scores

	Normal group	FHI group	*P* value
Pre-op mHHS	69.9 ± 8.8	65.2 ± 6.1	0.56
Post-op mHHS	81.7 ± 9.1	70 ± 10.9	**0.014**
Pre-op iHOT-12	44.8 ± 15.1	41.3 ± 11.1	0.65
Post-op iHOT-12	69.5 ± 17.5	38.5 ± 15.3	**0.002**
Pre-op VAS	3.5 ± 1.4	4.2 ± 1.3	0.33
Post-op VAS	1.2 ± 1.1	4.9 ± 1.9	**<0.001**

Data are mean ± SD. FHI, femoral head infarction. mHHS, modified Harris Hip Score; iHOT-12, International Hip Outcome Tool; VAS visual analog scale

In the normal group, 21 patients (70.0%) surpassed the MCID, and 26 patients (86.7%) achieved the PASS for mHHS. Additionally, in the normal group, 21 patients (70.0%) surpassed the MCID, and 16 patients (53.3%) achieved the PASS for iHOT-12. On the other hand, in the FHI group, only 3 patients (30%) surpassed the MCID and achieved the PASS for mHHS, while no patients surpassed the MCID or achieved the PASS for iHOT-12. The proportion of patients who achieved the MCID or the PASS in the normal group was significantly higher than that in the FHI group (*P* < 0.05).

## Discussion

In this study, we found that 2.7% of patients had FHI after hip arthroscopy for treatment of FAI. Patients in FHI group had significantly lower BMI than patients without FHI (*P* = 0.008). Patients in the FHI group did not show significant improvements in postoperative mHHS, iHOT-12, or VAS scores (*P* > 0.05). There was no significant difference in the preoperative patient-reported outcome scores between the normal group and the FHI group (*P* > 0.05). However, patients in the FHI group demonstrated significantly lower postoperative mHHS and iHOT-12 scores, along with higher VAS scores (*P* < 0.05). The proportion of patients who achieved the MCID or the PASS in the normal group was significantly higher than that in the FHI group (*P* < 0.05).

As the number of hip arthroscopy procedures increases, there is a corresponding rise in the frequency of observed and reported complications. These may encompass septic arthritis, neuropraxia, hemorrhage, bursitis, instrument breakage, chondral and labral damage, as well as fluid extravasations [9, 14–16]. Avascular necrosis is commonly regarded as potential complications in the excision of an impingement lesion during hip arthroscopy. Sampson et al. [18] reported one case of avascular necrosis after hip arthroscopy resulting in permanent damage. Sener et al. [25] also reported one case of avascular necrosis in a 61-year-old woman who underwent hip arthroscopy for a labral tear. There are no comparable reports of avascular necrosis in subsequent studies. However, it is crucial for the surgeon to be mindful of the retinacular vessels, which serve as the primary blood supply to the femoral head and run along the lateral femoral neck, as they are susceptible to intra-operative damage [26, 27]. The imaging manifestations of FHI and ischemic necrosis were different. The location of the infarction in this study were all located on the anterior lateral side of the femoral head and neck. We thought that the potential cause of FHI could be damage to the retinacular vessels. Further studies are warranted to explore the underlying reasons for FHI.

In this study, we found that patients with lower BMI are at a higher risk of developing postoperative FHI. As perivascular adipose tissue had been reported as a protection factor by releasing many vasoactive factors eliciting a net anticontractile and anti-inflammatory paracrine effect [28], one potential explanation was that patients with lower BMI may have less protection from fat on blood vessels, making them more prone to injury during surgery. Another potential explanation was that patients with low BMI had effective circulating blood volume because low BMI has been associated with low blood pressure and skin blood flow [29, 30]. However, this is just a conjecture, we need evidence to prove it in the future. We also found that the clinical outcomes for patients with FHI are poor, with no significant improvement in patient-reported outcomes observed. The postoperative mHHS, iHOT-12, and VAS scores of patients with FHI were significantly lower compared to those without FHI. This highlights the significant impact of FHI on clinical outcomes. It is imperative that we allocate more attention to FHI and take this factor into consideration when evaluating patients with suboptimal postoperative results.

Three patients underwent subsequent continuous MRI follow-up. It appears that one patient’s FHI has healed over time. The FHI of the other two patients has shown improvement, although it still persists. We hypothesized that infarction may improve due to vascular regeneration and compensation mechanisms. The retinacular vessels branch from the medial (superior and inferior retinacular arteries) and lateral (anterior retinacular artery) femoral circumflex arteries to penetrate the capsule [31, 32]. The anterior retinacular artery (ARA) regresses to supply blood to the anterior metaphyseal area, whereas the superior and inferior retinacular arteries (IRA) extend along the neck and serve as the primary blood supply to the femoral head [32, 33]. Conventionally, the superior retinacular artery (SRA) is considered to be the primary blood supply to the femoral head [34, 35], while the IRA has received a more minor consideration over the years [33]. A study using cadaveric specimens demonstrated through quantitative MRI that the IRA provides significant perfusion to the entire femoral head [36]. Liu et al. investigated the blood supply in patients with osteonecrosis of the femoral head using digital subtraction angiography and reported the SRA supports the blood supply in the caudal-lateral region and the IRA provide about 1/4 blood supply in the inferior-lateral head [37]. We prioritize the preservation of the SRA during arthroscopic surgery. However, there is a risk of neglecting the protection of inferior retinacula of Weitbrecht during femoral osteoplasty, potentially resulting in IRA damage. Besides, ARA is also a significant blood vessel for the femoral head; [38, 39] however, it has been relatively underemphasized. Overly distal femoral osteoplasty can potentially harm the ARA and result in FHI. Meanwhile, previous study also reported traction impairs blood perfusion to the femoral head [40]. Hence, we recommend focusing on safeguarding the inferior retinacula of Weitbrecht during hip arthroscopic surgery and avoiding excessive surgical traction and overly distal femoral osteoplasty to prevent potential damage to the ARA. Careful handling during surgery and timely postoperative review will be helpful. It is important to mention that since we have focused on protecting inferior retinacula of Weitbrecht and avoid overly distal femoral osteoplasty, there have been no instances of FHI. However, further research is required to substantiate this observation.

This study had several limitations. Firstly, as FHI is a rare postoperative complication, potential selection bias was neglectable. Secondly, the specific reasons for FHI remain unclear, necessitating further research to elucidate the underlying factors. Thirdly, only one patient underwent MRI reconstruction due to the complexity of the technology involved in MRI reconstruction. The purpose of this singular case reconstruction was to visually illustrate the location and extent of the FHIs in a more intuitive manner. Fourth, some patients have not undergone MRI follow-up, which could result in an increased calculated incidence of FHI. Moreover, although PROs and postoperative MRI were all conducted at least one year after surgery, their follow-up timelines were not synchronized. Furthermore, the number of patients with FHI was relatively small. In the future, larger sample MRI follow-up studies are needed to gain a more comprehensive understanding of this complication.

## Conclusion

Our study demonstrated that FHI could be a rare complication following hip arthroscopy. Patients with postoperative FHI had lower BMI. The clinical outcomes for patients with FHI are poor, with no significant improvement in patient-reported outcomes observed.

## Data Availability

All relevant data supporting the conclusions are included within the article and tables. The datasets used and/or analyzed during the current study available from the corresponding author on reasonable request.

## Abbreviations

MRI: Magnetic resonance imaging
FHI: Femoral head infarction
FAI: Femoroacetabular impingement
CT: Computer tomography
BMI: Body mass index
LCEA: Lateral center-edge angle
3D: Three-dimensional
PROs: Patient-reported outcomes
VAS: Visual analog scale
iHOT-12: International Hip Outcome Tool
mHHS: Modified Harris Hip Score
MCID: Minimal clinically important difference
PASS: Patient acceptable symptom state
SD: Standard deviation
ARA: Anterior retinacular artery
IRA: Inferior retinacular arteries
SRA: Superior retinacular artery
